# Positive Bacterial Culture among Lower Respiratory Tract Specimens of Patients in a Tertiary Care Centre: A Descriptive Cross-sectional Study

**DOI:** 10.31729/jnma.7219

**Published:** 2022-04-30

**Authors:** Shusila Khadka, Achut Barakoti, Ram Prasad Adhikari, Laxmi Kant Khanal, Jyotshna Sapkota

**Affiliations:** 1 Department of Microbiology, Nepal Medical College and Teaching Hospital, Jorpati, Attarkhel, Kathmandu Nepal

**Keywords:** *antibiotic resistance*, *gram negative bacteria*, *respiratory tract infections*

## Abstract

**Introduction:**

Lower respiratory tract infection accounts for a great burden of disease worldwide. The problem has further increased due to increasing antimicrobial resistance. This study was done to find out prevalence of positive bacterial culture among lower respiratory tract specimens of patients in a tertiary care centre.

**Methods:**

A descriptive cross-sectional study was done in the Laboratory of Clinical Microbiology in a tertiary care centre from May, 2021 to October, 2021. Ethical approval was received from the Institutional Review Committee (Reference number: 045-077/078). A total of 635 specimens were collected by convenience sampling. The specimens were cultured as per standard microbiological techniques. Antibiotic susceptibility was performed following Clinical and Laboratory Standards Institute (2020) guidelines. Microsoft Excel was used for data entry and analysis. Point estimate at 95% Confidence Interval was calculated along with frequency and proportion.

**Results:**

Among the 635 lower respiratory specimens, 112 (17.63%) (111.97 to 112.03 at 95% Confidence Interval) showed positive bacterial culture. *Klebsiella pneumoniae* 44 (37.93%) was the commonest isolate followed by *Acinetobacter calcoaceticus baumannii complex* 34 (29.31%).

**Conclusions:**

The prevalence of positive bacterial culture among lower respiratory specimens was lower when compared to other studies done in similar settings.

## INTRODUCTION

Lower Respiratory Tract Infection (LRTI) is the inflammation of the respiratory tract, which includes bronchitis, bronchiectasis, bronchiolitis, emphysema, lung abscess, pleural effusion, and pneumonia.^1^ It accounts for more than one-third of the reported deaths in Southeast Asia.^2^ The causative agents could be bacterial, viral or fungal. Among the bacterial causes, *Klebsiella pneumoniae, Pseudomonas aeruginosa, Acinetobacter calcoaceticus, Streptococcus pneumoniae, Staphylococcus aureus* are the common agents to cause lower respiratory tract infections.^3^

The emergence of Antimicrobial Resistance (AMR) is a major global health problem.^4^ Monitoring the antimicrobial resistance patterns of the etiological agents is needed not only to guide the clinicians when managing cases requiring antibiotic therapy but also to surveil the trend of these infections.^5,6^

The objective of this study is to find out positive bacterial culture among lower respiratory tract specimens of patients in a tertiary care centre.

## METHODS

This was a descriptive cross-sectional study done in the Laboratory of Clinical Microbiology of Nepal Medical College and Teaching Hospital for a period of six months, from May, 2021 to October, 2021. Ethical approval was received from the Institutional Review Committee (Reference number: 045-077/078). All the lower respiratory specimens (sputum, pleural fluid, bronchoalveolar lavage, endotracheal secretions) received for culture and sensitivity were included and duplicated lower respiratory specimens were excluded.

The sample size was calculated using the formula,

n = (Z^2^ × p × q) / e^2^

  = (1.96^2^ × 0.3042 × 0.6958) / 0.04^2^

  = 509

Where,

n = minimum required sample size
Z = 1.96 at 95% Confidence Interval (CI)
p = prevalence of positive bacterial culture, 30.42%^6^
q = 1-p
e = margin of error, 4%

Adding 10% to adress the non-response rate, we got a 565 sample size. However, total sample size of 635 was taken. The culture of the sample and identification of the organism was done following standard microbiological techniques by the American Society for Microbiology (ASM).^8^ Samples were cultured on Chocolate Agar (CA), 5% Blood Agar (BA), and MacConkey Agar (MA). On the CA plate, bacitracin (10 units) and optochin disk (5 mcg) were placed at primary and secondary inoculation to screen for *Haemophilus influenzae* and *Streptococcus pneumoniae* respectively. The CA and BA plates were incubated in CO_2_ incubator (10% CO_2_) at 37°C for 24 hours while MA plate was incubated at 37°C for 24 hours in aerobic atmosphere. Identification of isolated organisms included morphological study of colonies, Gram staining, and a battery of biochemical tests as required. Antibiotic susceptibility testing (AST) was done by Kirby-Bauer disc diffusion method following Clinical and Laboratory Standards Institute (CLSI).^9^

Microsoft Excel was used for data entry and analysis. Point estimate at 95% Confidence Interval was calculated along with frequency and proportion.

## RESULTS

**Figure 1:**
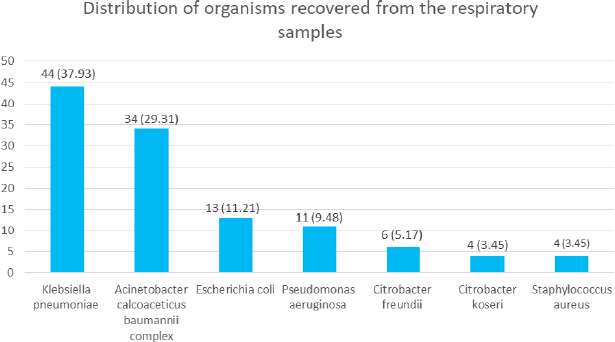
Distribution of the bacterial isolates in the respiratory sample (n= 112).

Among the total 635 specimens processed, the prevalence of positive bacterial cultures was seen in 112 (17.63%) samples (111.97-112.03 at 95% Confidence Interval). There were 71 (63.39%) sputum sample, 22 (19.64%) tracheal aspirates, 16 (14.28%) endotracheal tubes, 2 (1.78%) pleural fluid, and 1 (0.89%) bronchoalveolar lavage. A total of 108 (96.42%) samples showed significant monomicrobial growth while 4 (3.57%) of the samples showed significant polymicrobial growth (growth of two different microbes). Among the total bacterial isolates of 116, 112 (96.55%) were gram negative whereas 4 (3.44%) were gram positive. *Klebsiella pneumoniae* 44 (37.93%) was the most predominant organism followed by *Acinetobacter calcoaceticus baumannii* (ACB) complex 34 (29.31%). The other isolates included *Escherichia coli, Pseudomonas aeruginosa, Citrobacter species, Staphylococcus aureus* (Figure 1).

The overall susceptibility of gram-negative bacteria was highest towards carbapenems (imipenem, meropenem) with resistance rates of 23 (20.53%). A high rate of resistance was seen among the isolates of the ACB complex against the antimicrobial agents tested (Table 1).

**Table 1:** Resistance pattern of the gram-negative bacterial isolates (n= 112).

	Meropenem/ Imipenem n (%)	Cefotaxime/ Cefixime/Ceftazidime n (%)	Piperacillin-Tazobactam n (%)	Amikacin n (%)	Levofloxacin n (%)	Cotrimoxazole n (%)	Tigecycline n (%)
*Klebsiella pneumonia* (n= 44)	10 (22.72)	33 (75)	17 (38.63)	11 (25)	19 (43)	28 (63.63)	8 (18.18)
*Acinetobacter calcoaceticus baumannii complex* (n= 34)	11 (32.35)	34 (100)	29 (85.29)	29 (85.29)	28 (82.35)	30 (88.23)	3 (8.82)
*Escherichia coli* (n= 13)	1 (7.69)	8(61.53)	2 (15.38)	2 (15.38)	8 (61.53)	4 (30.76)	2 (15.38)
*Citrobacter freundii* (n= 6)	1 (16.67)	5 (83.33)	3 (50)	3 (50)	3 (50)	4 (66.67)	1 (16.67)
Citrobacter koseri (n= 4)	-	2 (50)	-	-	1 (25)	1 (25)	-
Pseudomonas aeruginosa^*^ (n= 11)	-	3 (27.27)	3 (27.27)	-	3 (27.27)	11 (100)	11 (100)
Total	23 (20.53)	85 (75.89)	51 (45.53)	45 (40.17)	62 (55.35)	78 (69.64)	25 (22.32)

* The resistance pattern mentioned in the above table in case of *Pseudomonas aeruginosa* for cephalosporins is only for ceftazidime.

The highest susceptibility of the ACB complex was towards tigecycline. Gram-positive organisms *(Staphylococcus aureus)* exhibited the highest susceptibility toward amikacin (Table 2).

**Table 2:** Resistance pattern of gram-positive isolates (n= 4).

Antibiotic class	Antibiotics	Resistant grampositive bacteria n (%)
Penicillin	Cloxacillin	3 (75)
Cephalosporin	Cephalexin	3 (75)
	Cefoxitin	3 (75)
Quinolones	Levofloxacin	2 (50)
Aminoglycoside	Amikacin	-
Sulfonamides	Cotrimoxazole	1 (25)
Macrolide	Erythromycin	3 (75)
Lincosamide	Clindamycin	3 (75)

Three (75%) of the 4 *Staphylococcus aureus* were Methicillin-Resistant *Staphylococcus aureus* (MRSA).

## DISCUSSION

Out of 635 samples, 116 organisms were recovered from 112 (17.6%) culture-positive samples, leaving a great number of negative results 523 (82.4%), which could be attributed to another aetiology such as viral, fungal, atypical microorganisms or may be due to prior use of antibiotics. Among the total isolates of 116, gram-negative bacteria were more predominant 112 (96.6%) as compared to gram-positive 4 (3.4%). A similar finding was present in a study done in Kerala with a culture positivity of 26.3% and gram-negative predominance (84.7%).^1^ Another study was done in Nepal also shows cultural positivity of 30.2% with gram-negative predominance.^10^

In this study, *Klebsiella pneumoniae* (37.9%) was the most common isolate followed by ACB complex (29.3%). The other isolates included *Escherichia coli, Pseudomonas aeruginosa, Citrobacter species, Staphylococcus aureus.* A study done in Italy showed that gram-negative isolates were more common in the lower respiratory sample (72.5%) and the common isolates were *Acinetobacter baumannii* (18.6%), *Staphylococcus aureus* (15.2%), *Pseudomonas* *aeruginosa* (14.2%), and *Klebsiella pneumoniae* (10.9%).^11^ A study done in China also showed gramnegative predominance with *Pseudomonas aeruginosa* as the commonest cause.^12^ In a similar study done in Sri Lanka, *Pseudomonas aeruginosa* was the most common isolate followed by *Klebsiella pneumoniae*.^13^ In a study conducted in Western Rajasthan, India, *Pseudomonas species* was the commonest isolate (31%), followed by *Klebsiella pneumoniae* (21.3%) and *Acinetobacter species?^4^* Another study in Nepal showed *Klebsiella pneumoniae* was the most common isolate followed by *Pseudomonas aeruginosa, Escherichia coli, Acinetobacter,* and *Staphylococcus aureus.^7^* So, it seems that gram-negative bacteria are more common in causing respiratory tract infections these days with *Klebsiella pneumoniae, Acinetobacter baumannii, Pseudomonas aeruginosa* being the most common isolates. However, the etiological agents of respiratory infections and their antibiotics susceptibility can vary from one area to another.^4^

Gram-negative bacteria were the predominant isolates in this study. Both gram-negative and gram-positive isolates demonstrated a high rate of resistance to widely used antibiotics tested. Gram-positive organisms *(Staphylococcus aureus*) exhibited resistance to cloxacillin (75%), cefoxitin (75%), erythromycin (75%), and clindamycin (75%) (Table 2). Three out of the 4 *Staphylococcus aureus* (75%) were MRSA. In a study, a total of 92.9% *Staphylococcus aureus* were MRSA and Ciprofloxacin resistant, 64.3% were resistant to cotrimoxazole, and 28.6% were resistant to amikacin.^15^

The overall susceptibility of gram-negative bacteria was highest towards carbapenems (imipenem, meropenem) with resistance rates of 20.5% (Table 2). This is in concordance with another study that reported that gram-negative bacteria have a higher sensitivity to carbapenems.^10^ High rate of resistance of the gramnegative isolates was observed towards ampicillin, cephalosporins, cotrimoxazole, and levofloxacin. The gram-negative isolates were resistant to most of the antibiotics tested. ACB complex exhibited a high rate of resistance to most of the antibiotics tested (Table 1). It was resistant to most antibiotics like cefixime (100%), piperacillin-tazobactam (85.3%), amikacin (85.3%), and levofloxacin (82.4%). Similar studies have also found *Acinetobacter* to be resistant to multidrug like cefixime, ciprofloxacin, and azithromycin.^16^

Another study was done in India and also has shown high resistance of *Acinetobacter* to cephalosporins, piperacillin-tazobactam, carbapenems, and most other antibiotics.^17^ In this study, *Acinetobacter species* had a higher sensitivity of 91.2% to tigecycline. A study done in Korea has shown that tigecycline is well tolerated and the clinical outcome of tigecycline therapy is comparable to that of colistin therapy for multi-drug resistant (MDR) *Acinetobacter species*. So, tigecycline could be used as an alternate for the treatment of MDR *Acinetobacter species.^18^* The increasing antibiotic resistance is mainly attributed to the misuse of antibiotics so they should be prescribed judiciously.^4^

This study had some limitations. The descriptive nature of the study couldn't establish causality and association between the study variables. Further, a study in a larger population could increase the generalizability of the findings.

## CONCLUSIONS

Our study showed that the prevalence of positive bacterial culture among lower respiratory specimens was lower when compared to other studies done in similar settings. In this study, a high level of resistance to antibiotics was observed among the respiratory isolates. So, there is a need to perform antibiotic susceptibility testing before empirical therapy. The dissemination of the data of this type of study would be useful for the clinicians to make a proper choice of empirical antibiotics when managing cases of lower respiratory infections requiring antibiotic therapy.
